# Quantum chemistry rules retinoid biology

**DOI:** 10.1038/s42003-023-04602-x

**Published:** 2023-02-28

**Authors:** Ulrich Hammerling, Youn-Kyung Kim, Loredana Quadro

**Affiliations:** Department of Food Science, Rutgers Center for Lipid Research and Institute of Food Nutrition and Health, Rutgers University, New Brunswick, NJ USA

**Keywords:** Lipids, Energy metabolism

## Abstract

This Perspective discusses how retinol catalyzes resonance energy transfer (RET) reactions pivotally important for mitochondrial energy homeostasis by protein kinase C δ (PKCδ). PKCδ signals to the pyruvate dehydrogenase complex, controlling oxidative phosphorylation. The PKCδ-retinol complex reversibly responds to the redox potential of cytochrome c, that changes with the electron transfer chain workload. In contrast, the natural retinoid anhydroretinol irreversibly activates PKCδ. Its elongated conjugated-double-bond system limits the energy quantum absorbed by RET. Consequently, while capable of triggering the exergonic activating pathway, anhydroretinol fails to activate the endergonic silencing path, trapping PKCδ in the ON position and causing harmful levels of reactive oxygen species. However, physiological retinol levels displace anhydroretinol, buffer cyotoxicity and potentially render anhydroretinol useful for rapid energy generation. Intriguingly, apocarotenoids, the primary products of the mitochondrial β-carotene,9'-10'-oxygenase, have all the anhydroretinol-like features, including modulation of energy homeostasis. We predict significant conceptual advances to stem from further understanding of the retinoid-catalyzed RET.

## Abstract (web summary)

This Perspective explores retinol’s resonance energy transfer (RET) capabilities for reversible activation of PKC delta, so pivotal for OXPHOS control, and illuminates novel aspects of retinoid biology.

The pyruvate dehydrogenase complex (PDHC) plays a central role in the regulation of fuel-flux and -choice^1^. This enzyme is positively controlled by two phosphatases, pyruvate dehydrogenase phosphatase PDP 1 and 2, and negatively by four kinases, pyruvate dehydrogenase kinase PDK 1–4^2^. While the controls of these PDKs and PDPs are manifold, information regarding pertinent upstream signal systems is still incomplete. We found that the serine-threonine kinase, protein kinase Cδ (PKCδ) functions as a controller of the PDHC^3^. PKCδ, located in the intermembrane space of mitochondria, targets the PDK2 enzyme indirectly *via* a yet to be defined phosphatase, leading to de-phosphorylation and hence inactivation of PDK2^4^ (Fig. 1a). As the negative impact of PDK2 on PDHC is waning, PDP’s positive influence on the PDHC is increasing the flow of glucose-derived acetyl-CoA into the Krebs cycle, consequently upregulating oxidative phosphorylation (OXPHOS) (Fig. 1a). Our key postulate is that the PKCδ signal system be reversible, as unabridged PKCδ signaling leads to production of harmful levels of reactive oxygen species (ROS) by an overtaxed electron transfer chain^5^.

**Fig. 1:**
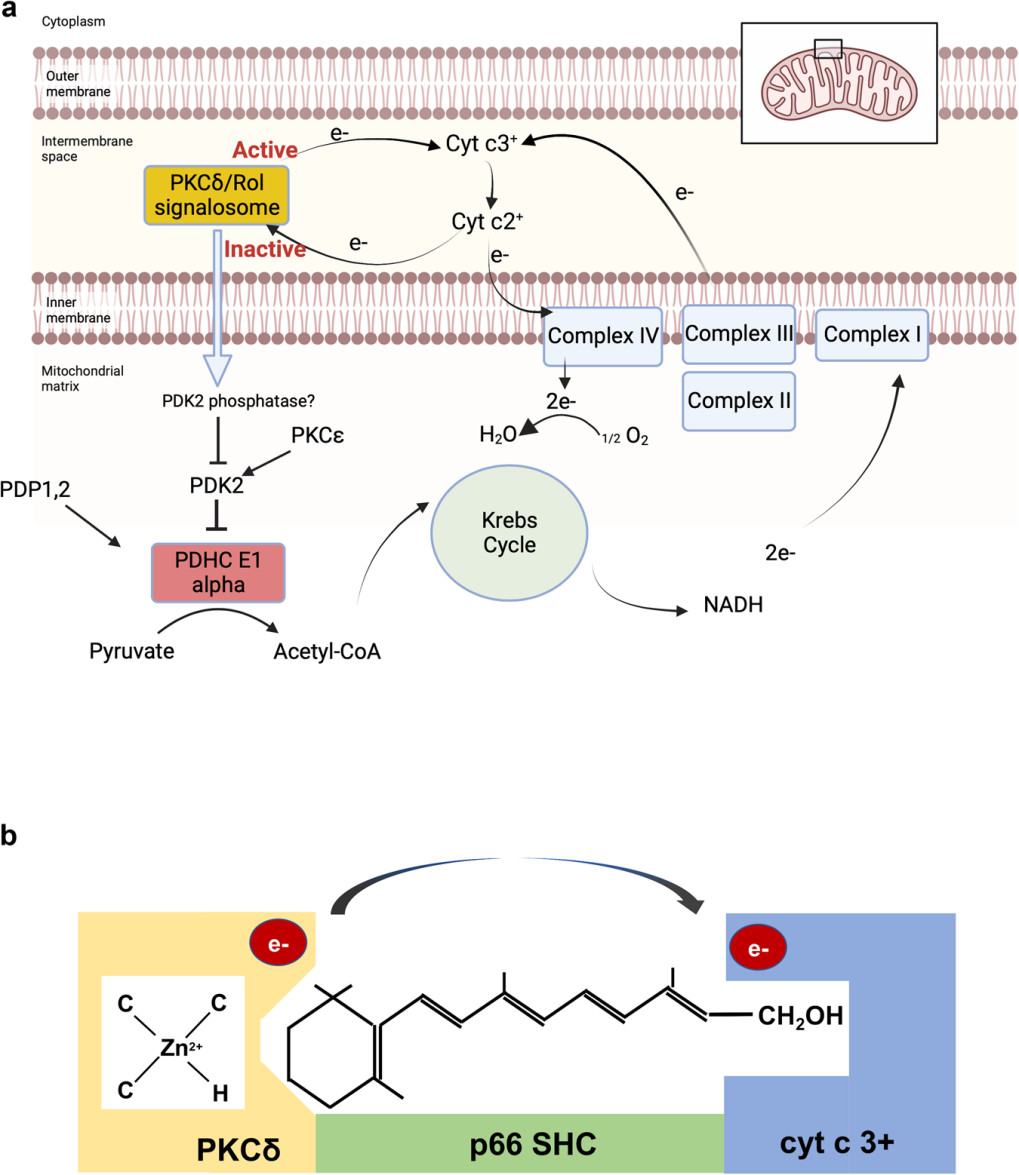
Schematic of the PKCδ signalosome and its reversible redox regulation. **a** Reversible redox regulation of PKCδ. The PKCδ/Retinol (Rol) signalosome, located in the mitochondrial intermembrane space, comprises PKCδ, the signal adapter p66Shc, cytochrome c (cyt c) and all-trans retinol (See Fig. 1b). Activation of PKCδ is initiated by site-selective oxidation of specific cysteines in its activation domain, a process involving the retinol-catalyzed transfer of an electron pair from PKCδ to signalosome-associated cyt c3 + . The PKCδ signal to PDH is amplified by an enzyme cascade involving pyruvate dehydrogenase kinase 2 (PDK2)-phosphatase (yet to be identified) that inactivates PDK2. In absence of PDK2, the activity of pyruvate dehydrogenase phosphatases (PDP1,2) effectively dephosphorylates, and thereby activates, the E1 alpha regulatory domain of PDHC, leading to enhanced production of Acetyl-CoA from Pyruvate. Acetyl-CoA enters the Krebs cycle, driving the generation of NADH. The high-potential electron of NADH passes through the electron transfer chain (ETC). Redox activation of PKCδ is reversible. During periods of high electron flux cyt 2+ accumulates in the intermembrane space gradually replacing signalosome-associated oxidized cyt c, bringing about a reversal of redox polarity, and thus enabling the passage of electrons back to oxidized PKCδ. As the reduced, inactive form of PKCδ is restored, signaling to PDHC is weakened and consequently fuel flux diminishes, reducing the risk of ETC overload. Note that PKCε is also known to activate PDK2 counteracting the action of PKCδ^28^. This activity is not discussed herein. **b** Composition of the PKCδ signalosome. The PKCδ signal complex comprises the protein kinase Cδ, the adapter protein Src homologous-collagen homolog (p66Shc), cytochrome c (cyt c) and all-trans-retinol. PKCδ binds p66Shc in such a manner that it juxtaposes cytochrome c, also bound to the p66Shc platform. Retinol binds PKCδ on a site within the PKCδ activation domain in close proximity of its landmark zinc-coordinated structure. A conserved tryptophane residue enables resonance energy transfer^18^ from PKCδ to retinol. The task of retinol is believed to catalyze electron- (or rather energy-) transfer to cyt c 3 + , initiating an unfolding process that yields the active form of the enzyme. Panel A was created with BioRender.com.

It is noteworthy that, in neurons, mitochondrial PKCδ signals not only to PDHC but also to dynamin-related protein 1 (DRP-1)^6^. Phosphorylation by PKCδ of a specific serine moiety of DRP-1 (in mice, Ser 635) enables binding of FIS-1 to start assembly of the fusion/fission complex which controls mitochondrial integrity^7^. It is not clear whether a single pool of retinol and cytochrome c-dependent PKCδ, reigns over both PDHC and DRP-1 or whether two independently acting pools reside in mitochondria. It seems however no coincidence that unchecked activation of PDHC by PKCδ results in progressive oxidative stress, the very same condition that triggers mitochondrial fission.

Unlike cytosolic PKCδ and other isoforms that use di-acyl-glycerol as a second messenger, the mitochondrial PKCδ activity is controlled by a redox mechanism. This is accomplished by an associated oxido-reductase complex, comprising p66Shc, a signal adapter protein, cytochrome c (cyt c) and vitamin A alcohol (retinol)^4^ (Fig. 1b). Not strictly an oxido-reductase per se, cyt c links the PKCδ signalosome to cytochrome c reductase and cytochrome c oxidase complexes, the two most powerful redox enzymes of mitochondria. We postulate that, owing to higher efficiency of the cyt c reductase over that of the oxidase, reduced cyt c accumulates at high electron flux, whereas oxidized cyt c prevails at low flux. By reading the changes in the ratio of oxidized to reduced cyt c, and responding either positively (at high cyt c^ox^: cyt c^red^) or negatively (at the inverse ratio), PKCδ adjusts the fuel flux entering into the Krebs cycle up- or downwards (Fig. 1a). In this manner PKCδ would monitor the workload of the electron-transfer chain with the goal of keeping OXPHOS within safe margins.

While the PKCδ signalosome is of prime importance to lymphocytes^8^, we also showed that lack of PKCδ impaired normal functions of liver, adipose and muscle^9^. Furthermore, other reports have indicated that mitochondria isolated from spleen, liver, brain, heart use PKCδ signaling for fuel flux regulation^10^. Thus, we favor the hypothesis that PKCδ has similar functions in multiple tissues/organs and perhaps at different stages of the life cycle.

A key feature that explains how the redox-mediated ON-OFF switching of the PKCδ works is the zinc-finger-like structure embedded in the kinase activation domain^11^. The prototype of such a zinc-finger exists in bacteria, often referred to as redox-sensitive reversible switch^12^. The bacterial HSP33 chaperone contains a conserved string of four cysteine moieties spatially arranged into a perfect tetrahedron, with a zinc-ion at its center^13^. This zinc-coordinated structure is stable as long as reducing conditions prevail. However, in response to oxidizing conditions it undergoes a rapid structural, and subsequently functional remodeling that yields the active chaperone^14^. While the mammalian PKCδ ortholog is considerably more complex, containing for instance two intertwined zinc coordination centers, each formed by three cysteines and one histidine^11^, it performs a similar function, namely stabilization of a rigid, tertiary structure under reducing conditions. The strong electron negativity of the zinc-finger should confer rapid responsiveness to oxidizing agents that activate this kinase^15^. In contrast to bacteria, however, the mammalian zinc-finger requires site-specific cysteine oxidation by an oxido-reductase because soluble oxidizers, such as hydrogen peroxide, invariably cause irreversible structural damage to the protein^16^, although *de facto* transiently activating PKCδ^17^.

Oxidative kinase activation entails the successive transfer of at least 2 electrons from the zinc-finger cysteines to ferricytochrome c and onwards to molecular oxygen *via* cyt c oxidase (Fig. 2). This electron transfer is facilitated by juxtaposing PKCδ to cyt c, on the p66Shc platform^4^ (Fig. 1b). Despite the close proximity, we assume that the VanDerWaal’s contact between PKCδ and cyt c is not achieved, hence requiring an electronic bridge for expediency. This is provided by retinol (vitamin A alcohol) which binds PKCδ at a dedicated site near the zinc-finger domain (C1B domain)^18^. We propose that arranging the PKCδ-vitamin A-p66Shc-cyt c components into a supercomplex essentially creates an auxiliary electron transfer chain which allows rapid control of the PDHC in both directions.

**Fig. 2:**
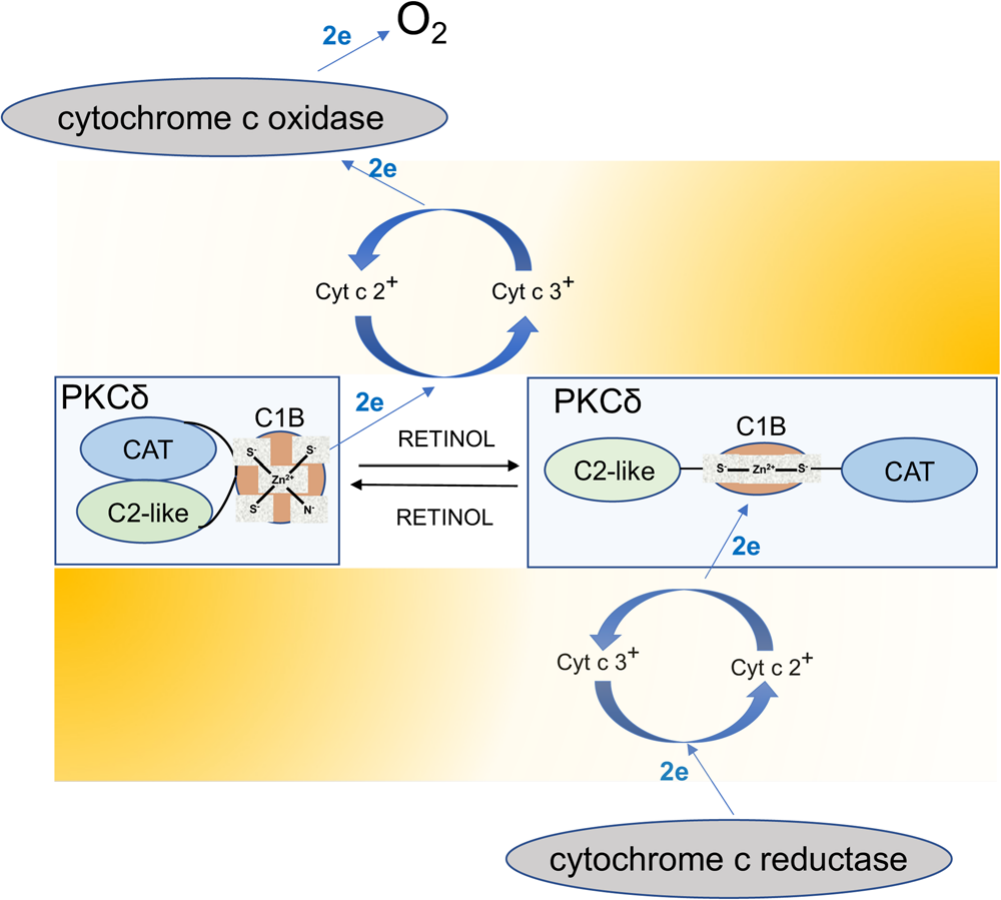
A binary switch controls the activation/inactivation cycle of PKCδ. Left pane shows autoinhibited PKCδ enzyme. Driven by its strong electro-negative redox potential (symbolized by a redox gradient in yellow), and catalyzed by retinol, a pair of electrons originating from the zinc-finger domain (C1B) passes to oxidized cytochrome cyt c3^+^ and onwards to cytochrome C oxidase and from there to O_2_ (left side of the figure). The loss of at least 2 negatively charged cysteinyl anions triggers the unfolding of the zinc-finger domain. In an ensuing multistep process, the autoinhibitory regulatory domain (C2-like) is withdrawn, uncovering functional sites of the catalytic domain (C1B), rendering PKCδ fully active. As the redox potential reverses polarity (right side of the figure), electrons donated by the electron transfer chain pass via cyt c 2^+^ to the cysteine-rich domain of PKCδ, restoring the zinc-finger structure. Our model suggests that the PKCδ activation/inactivation cycle is ratiometric, is closely linked to the workload of the ETC and thus adjusts fuel flux to energy demands, at the same time constraining the ETC from going over capacity.

Viewing electron transfer instead as energy transfer brings the function of the PKCδ signalosome into further focus. By binding the PKCδ zinc-finger domain, vitamin A is electronically coupled to a conserved tryptophane, enabling resonance energy transfer, as demonstrated in vitro by quenching of the intrinsic protein fluorescence with bound retinol elicited by UV irradiation of tryptophane at 290 nm^19^. In vivo the strong redox potential of the zinc-coordination center propels de-localized-electrons of retinol’s conjugated double-bond system to higher orbit(s). When returning to ground state, the energy is recovered to be used for the reduction of ferri- to ferro-cyt c (provided that ferri-cyt c+ is bound to its dedicated site on p66Shc^20^). The local structural change in the zinc-finger domain initiates large-scale allosteric remodeling of the protein to its enzymatically competent state. While we pinpointed resonance energy transfer from a conserved tryptophane of PKCδ to vitamin A as the likely entry into the auxiliary electron transfer chain (ETC) pathway^18^, we have not identified the efferent arm that mediates energy transfer from excited vitamin A to ferri-cyt c. However, the trail-blazing studies by the Pellicci’s group^20^ revealed that p66Shc, acting as an oxido-reductase-like enzyme, can greatly speed up the transfer of electrons from reduced cyt c to molecular oxygen in vitro, providing clues that p66Shc may function also in vivo as an important active component of energy transfer. These authors did not test energy transfer in the reverse direction, i.e., from a biologically relevant electron donor (such as excited vitamin A) to ferri-cyt c.

The transfer of 2 electrons from the resting PKCδ to ferri-cyt c is likely driven by the high-energy electrons of cysteinyl anions contained in the zinc-coordination center^11^. In this case, cyt c reduction is initiated when mitochondria are energized and one electron is entering the auxiliary electron transfer chain, resulting in the conversion of ferri- to ferro-cyt c. While theoretically capable of returning to PKCδ, a steep redox gradient propels the transferred electron forward to cyt c oxidase for permanent disposal to O_2_. Moving one electron creates a momentary cysteinyl radical which is promptly resolved by exporting the second electron to cyt c as well, changing in the process the PKCδ cysteinyl anions to disulfides (Fig. 2).

While nominal values for zinc-coordinated cysteines are not available, a comparable value for the reduced *versus* oxidized glutathione redox couple is nominally 240 millivolts^21^. Assuming a similar nominal value for zinc-coordinated cysteines suggests sufficient potency to reduce ferri- to ferro-cyt, the standard reduction potential of the cyt c redox couple being 220 millivolts^22^. In our model the reduction of ferri-cyt c proceeds through a retinol intermediate, suggesting that the redox potential of resting *versus* excited retinol should be in the 200 millivolts range as well, or even exceed this value.

Matching redox potentials among the components of the PKCδ electron transfer chain is a prerequisite for reversibility. In other words, the transfer of electrons from PKCδ to cyt c that leads to kinase activation is required also for the reverse direction, from reduced cyt c to oxidized PKCδ (Fig. 2). The newly reduced cysteinyl anions are now available for the reconstruction of the zinc-finger which constitutes an important step in the refolding of the PKCδ into its inactive form. This refolding process likely is not spontaneous, as during the preceding step of cysteine oxidation much energy is supposedly lost to enthalpy. Furthermore, the subsequent unfolding step engenders energy loss to entropy. The Law of Conservation of Energy stipulates that in a closed system, such as that of the zinc-coordination center, any energy transferred out must be resupplied to regain its original internal energy status. It is unclear how this is accomplished in cells, but ATP-driven protein chaperones are frequently employed for this purpose. Interestingly, the mammalian mtHSP70 chaperone system, a precise ortholog of the bacterial HSP70 chaperone that assists in the refolding of the HSP33 zinc-finger^12^, was long known to form a complex with p66Shc^23^, suggesting that mtHSP70 might be a constitutive component of the PKCδ signalosome as well. Notably, impairment of the mtHSP70 chaperone system was linked to diseases in humans^24^. As an ATP-driven chaperone mtHSP70, together with HSP60 co-chaperones, also orthologs of the bacterial co-chaperones DNAJ, DNAK, and GroEL^25^, are strong candidates for orchestrating the reconstruction of the globular, inactive, PKCδ molecule. The emerging model is that of a dynamic PKCδ signaling pathway that responds to cues received from the electron transfer chain (ETC) *via* the redox status of cyt c (Fig. 1a). In particular, a high ratio of ferri- to ferro-cyt c would signal to increase energy demands, and hence activate PDHC, whereas the inverse cyt c ratio would promote the attenuation of the PDHC output to countermand imminent ETC overload. Pegging the system input i.e., the workload of the ETC as reflected in the ratio of reduced to oxydized cyt c to the output (i.e., PKCδ signaling strength, PDHC activity and fuel flux) is the definition of ratiometric control. In our models, this correlation is inverse, ratiometric nonetheless.

The collapse of the zinc tetrahydral structure could be initiated: 1. by targeted oxidation of cysteine residues moiety^4^, 2. by random oxidation by reactive oxygen species^17^ and 3. by dislodging one or more cysteines by binding of di-acyl-glycerol to the activation domain^15^. However, Gopalakrishna et al. showed that reactive oxygen species, foremost H_2_O_2_, are deleterious to the PKCδ protein owing to random oxidation of cysteine moieties^16^. Thus, we favor a model of targeted oxidation of the PKCδ activation domains by oxido-reductases, such as the cytochrome c system.

As stated above the oxidation of PKCδ zinc-finger-associated cysteines to presumptive cystines, is an exergonic process driven by a descending cascade of redox intermediates. We stipulate that the redox potential of retinol, which holds a central position in this auxiliary ETC, must be high enough to catalyze both the forward and the reverse pathways. However, because the latter is an energetically uphill reaction, during the reverse reaction the excited retinol intermediate must also resupply the energy lost to enthalpy. These uneven requirements for forward *versus* reverse catalysis have important consequences when retinoids with lesser redox potential substitute for retinol. Two such retinoids are anhydroretinol, a natural derivative of retinol^26^, and fenretinide, a synthetic derivative of retinoic acid^27^. Both retinoids bind PKCδ with similar affinity as retinol, both compete retinol off its binding sites, and both serve as potent co-activators of the PKCδ signaling pathway^28^. However, we showed that in the presence of anhydroretinol or fenretinide, the activation of the PKCδ-PDHC signal path is associated with uncontrolled ROS release, leading to progressive cell damage and even cell death^29^. Why anhydroretinol and fenretinide are capable of catalyzing PKCδ activation, yet are cytotoxic, and how this differs from the non-toxic action of retinol, is not fully understood (Fig. 3). We propose that differences in their conjugated double bond systems, specifically in the lengths of polyenes, are responsible. As a rule, the quantum of energy that any polyene can absorb is inversely correlated to the polyene length^30^. Therefore, retinol can absorb a substantially higher energy quantum than anhydroretinol or fenretinide. Compared to the UV absorption spectrum of retinol (UV_max_ = 325 nm), those of anhydroretinol and fenretinide (UV_max_ = 346 and 362, respectively) are red-shifted by *ca* 20 nm. The redox potentials acquired by anhydroretinol and fenretinide in their excited states might therefore be sufficient to catalyze the exergonic oxidation of PKCδ, thereby activating this enzyme, but too low for the chemical reduction path that silences the active PKCδ. In consequence, PKCδ might become trapped in its open, active conformation. A precedent of such a quantum trap is the xanthophyll cycle that controls excessive photon flux in photosynthesis^31^, not unlike the retinol-mediated control of the ETC by the PKCδ signalosome. Chronic PKCδ signaling is well-known to result in damaging levels of oxidative stress. For instance, the transgenic expression of constitutively active PKCδ in mitochondria^5^, the near-irreversible activation by phorbol ester^32^, even the natural overexpression of PKCδ in C57BL/6 mice^33^, or the exposure to anhydroretinol^29^, all promote a pathology marked by redox overload.

**Fig. 3:**
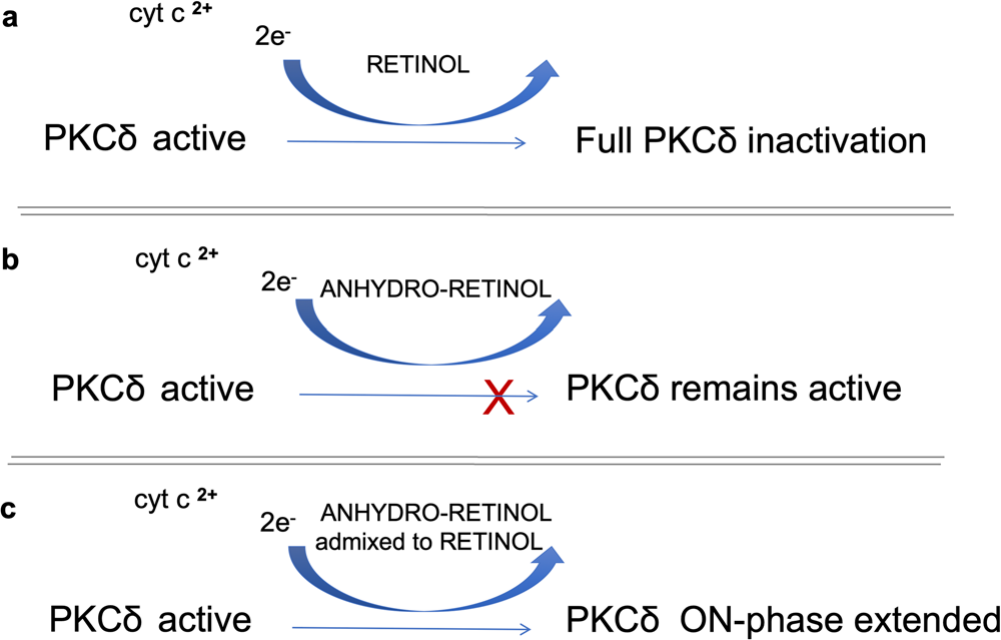
Structural differences in the conjugated double bond systems of retinoids exert dramatically different effects on PKCδ. Retinol serves as electronic conduit that facilitates the bidirectional electron transfer between PKCδ and cyt c. By contrast retinoids with extended polyene systems, such as anhydroretinol, allow electron transfer in the forward, but not reverse direction, leading to redox stress. Shown are responses to different retinoids in the PKCδ inactivation pathway. **a** In its activated (oxidized) state PKCδ can receive electrons from cyt c 2 + , initiating the re-construction of the zinc-coordination center leading to full enzyme inactivation. **b** Due to its extended polyene system, anhydro-retinol (along with fenretinide) is purported to absorb a lesser quantum of energy than retinol, consequently failing to shut down PKCδ in a timely manner and leading to mitochondrial oxidative stress. **c** When admixed in small quantities to retinol, anhydro-retinol can transiently boost PKCδ signaling, resulting in increased fuel flux in mitochondria.

A question arises as to the physiological relevance of anhydroretinol. This retinoid is a natural derivative, is enzymatically produced from retinol by specific dehydratases, has a wide tissue distribution, and is evolutionarily conserved up to drosophila^34^, but so far, no function has been identified in vivo for anhydroretinol. Moreover, the propensity of anhydroretinol for damage to cultured cell is counterintuitive. Why would nature evolve an agent solely destined to wide-spread cytotoxicity? Or would the relevance lie in the ability to super-activate the PKCδ signal pathway? Whereas experiments that uncovered the potent capacity to produce oxidative stress were carried out in vitamin A deprived cell cultures^26^, the presence of physiological retinol levels might buffer the negative impact of anhydroretinol in vivo. Retinol has in fact been shown to reverse its toxicity^35^. Thus, the possibility should be entertained that anhydroretinol, when added in small doses to the PKCδ signal system in the presence of normal retinol levels might prolong the PKCδ- as well as the downstream PDHC-ON phases, thereby contributing a healthy momentary energy boost.

Anhydroretinol levels have not been systematically assessed in animals and humans under physiological conditions and/or specific disease states. Anhydroretinol was detected in various mouse and human cell lines^29^. In one report^35^, our lab used anhydroretinol as a model compound in our quest to understand the regulation of fuel flux *via* the PKCδ/PDHC signaling system. The lack of identification of the mammalian retinol-dehydratase, the presumed ortholog of the only known insect dehydratase^36^, has hampered studies of the biological relevance of anhydroretinol, which remains unaddressed. Intriguingly, mitochondria harbor in the form of the β-carotene 9'-10' oxygenase (BCO2) enzyme and its products, apo-carotenoids, a potential signaling system in search of a target. BCO2 is one of the two mammalian enzymes—the other being β-carotene 15-15' oxygenase (BCO1)—that cleave carotenoids in mammalian tissues^37^. Carotenoids are C40 isoprenoid pigments characterized by a conjugated double bond system and synthesized exclusively by plants and microorganisms where they aid in photosynthesis or provide the bright colors of nature, among other functions^38^. Mammals acquire carotenoids from the diet and use them for pigmentation, protection from light or oxidative stress. The cleavage products of carotenoids are called apocarotenoids, a term that includes retinoids as well^39^. Apocarotenoids are formed both in plants and mammalian tissues where they perform specific functions generally related to signaling pathways. The two mammalian carotenoid cleavage enzymes BCO1 and BCO2 differ in substrate specificity and cellular and sub-cellular localization and hence in their function. The cytoplasmic BCO1 is the main enzyme that cleaves provitamin A carotenoids, such as β-carotene, to yield vitamin A aldehyde (retinal or β-apo-15-carotenal)^37^. In contrast BCO2, which is localized in the inner membrane of the mitochondria^40^ and shows broad substrate specificity towards various carotenoids^41^, has been proposed to cleave carotenoids to protect the mitochondria from the toxic effects of elevated concentrations of these pigments^42^. BCO2 cleaves specifically at the 9′,10′ bond of the parent compound to produce apo-10′-carotenoids from various substrates^38^.

BCO1 and 2 are often expressed in the same organ although in different cell types and subcellular compartments^40^. Surprisingly, BCO2 seems to be the only cleavage enzyme expressed in the adult heart. Analysis of the expression of the *BCO2* gene through the human Heart Single Cell Atlas (www.heartcellatlas.org) revealed that this gene is highly abundant in the atrial and ventricular cardiomyocytes, while also expressed at lower levels in other cell types of this organ, including endothelial cells, fibroblasts, pericytes, and smooth muscle cells. The question arises as to what the true function of BCO2 is, at least in the heart. It must be clearly a critical one, given its abundance. Perhaps this is linked to the high energy requirements for this organ and to its responsiveness to changing energy demands through utilization of multiple sources of energy-providing substrates^43^. It is therefore intriguing that BCO2 has several features expected for a regulator of PKCδ, such as its select expression in the inner mitochondria membrane^40^ as well as the local generation of apocarotenoids like β-apo-10'-carotenal (from cleavage of β-carotene). Like retinol, this apocarotenoid should be able to bind PKCδ *via* the β-ionone ring and activate this kinase, but unlike retinol it might not catalyze the PKCδ silencing pathway, given the diminished capacity of resonance energy transfer inherent in its elongated conjugated double bond system compared to that of retinol. However, when buffered by the physiological levels of retinol in the healthy heart β-apo-10'-carotenal should be able to signal for increased momentary energy output, without causing undue oxidative stress. While decisive experimental support for our hypothesis is still lacking, BCO2 and its primary cleavage product deserve undivided attention.

Overall, the pleiotropic functions of PKCδ have been understudied and we expect this area of investigation to increase in the near future.
